# Correlation between CD105 expression and postoperative recurrence and metastasis of hepatocellular carcinoma

**DOI:** 10.1186/1471-2407-6-110

**Published:** 2006-05-02

**Authors:** Lian-yue Yang, Wei-qun Lu, Geng-wen Huang, Wei Wang

**Affiliations:** 1 Liver Cancer Laboratory, Department of Surgery, Xiangya Hospital, Central South University, Changsha 410008, Hunan Province, P.R, China

## Abstract

**Background:**

Angiogenesis is one of the mechanisms most critical to the postoperative recurrence and metastasis of hepatocellular carcinoma (HCC). Thus, finding the molecular markers associated with angiogenesis may help identify patients at increased risk for recurrence and metastasis of HCC. This study was designed to investigate whether CD105 or CD34 could serve as a valid prognostic marker in patients with HCC by determining if there is a correlation between CD105 or CD34 expression and postoperative recurrence or metastasis.

**Methods:**

Immunohistochemical staining for the CD105, CD34 and vascular endothelial growth factor (VEGF) antibodies was performed in 113 HCC tissue specimens containing paracarcinomatous tissue and in 14 normal liver tissue specimens. The quantitation of microvessels identified by anti-CD105 and anti-CD34 monoclonal antibodies and the semiquantitation of VEGF expression identified by anti-VEGF monoclonal antibody were analyzed in conjunction with the clinicopathological characteristics of the HCC and any available follow-up information about the patients from whom the specimens were obtained.

**Results:**

CD105 was not expressed in the vascular endothelial cells of any normal liver tissue or paracarcinomatous liver tissue but was expressed in the vascular endothelial cells of all HCC tissue. In contrast, CD34 was expressed in the vascular endothelial cells of normal liver tissue, paracarcinomatous tissue, and HCC tissue in the following proportions of specimens: 86.7%, 93.8%, and 100%, respectively. The microvascular densities (MVDs) of HCC determined by using an anti-CD105 mAb (CD105-MVD) and an anti-CD34 mAb (CD34-MVD), were 71.7 ± 8.3 (SD) and 106.3 ± 10.4 (SD), respectively. There was a significant correlation between CD105-MVD and CD34-MVD (*r *= 0.248, *P *= 0.021). Although CD34-MVD was significantly correlated with VEGF expression (*r *= 0.243, *P *= 0.024), CD105-MVD was more closely correlated (*r *= 0.300, *P= *0.005). The correlation between microscopic venous invasion and CD105-MVD, but not CD34-MVD, was also statistically significant (*r *= 0.254, *P *= 0.018). Univariate analysis showed that CD105-MVD was significantly correlated with the 2-year overall survival rate (*P *= 0.014); CD34-MVD was not (*P *= 0.601). Multivariate analysis confirmed that CD105-MVD was an independent prognostic factor and that CD34-MVD was not.

**Conclusion:**

The anti-CD105 mAb is an ideal instrument to quantify new microvessels in HCC as compared with anti-CD34 mAb. CD105-MVD as compared with CD34-MVD is relevant a significant and independent prognostic indicator for recurrence and metastasis in HCC patients.

## Background

Hepatocellular carcinoma (HCC) is one of the most common cancers worldwide, especially in China, where it has been ranked as the second leading cancer killer since the 1990s [1]. Although some advances have occurred in the diagnosis and treatment of HCC, the long-term outcome for affected patients is still very poor: the 5-year recurrence rate of HCC is as high as 50% to 60% [2]. Therefore, identifying the molecular markers that correlate with the recurrence or metastasis of HCC may help improve the outcome in these patients.

Angiogenesis is one of the mechanisms most critical to the postoperative recurrence and metastasis of HCC [3-5]. Thus, finding the molecular markers associated with angiogenesis may help identify patients at increased risk for recurrence and metastasis of HCC, and thus those who require more aggressive therapy and closer surveillance. To date, however, no such marker has been definitively identified. One potential marker is the degree of neovascularization (ie, the microvascular density [MVD]). Several researchers have demonstrated that the MVD, as evaluated by an anti-CD34 monoclonal antibody (mAb) (CD34-MVD), was closely correlated with the prognosis of HCC [6,7]. However, other investigators have not found any such correlation [8,9]. These conflicting results are most likely due to the different reactivities of the anti-endothelial cell antibodies used to stain the intratumoral microvessels [10].

For example, antibodies against pan-endothelial cells, such as the anti-CD34 mAb, react with not only newly formed vessels but also normal vessels trapped within tumor tissues [11,12]. In contrast, the anti-CD105 mAb preferentially reacts with the activated endothelial cells of angiogenic tissues, including tumors, but weakly or not at all with the endothelial cells of most normal tissues [10,12-23]. Thus, the detection of CD105 is likely to be useful not only for its prognostic value as a means to identify candidates for specific antiangiogenic therapy. In fact, some investigators have already demonstrated that the MVD of cells, determined with an anti-CD105 mAb (CD105-MVD) but not with an anti-CD34 mAb, correlates with prognosis in some other types of carcinoma, such as breast, renal cell, and colorectal carcinoma [24-29]. Thus, our study attempts to investigate a correlation between CD105-MVD and the postoperative recurrence or metastasis of HCC.

## Methods

### Patients and specimens

The study protocol was approved by the Ethics Committee of the Central South University. Tumor tissue specimens from 113 patients with HCC who underwent complete tumor resection without any preoperative therapy in Xiang Ya Hospital in China from 1994 to 1997 were retrospectively reviewed. All tumors were pathologically confirmed to be HCC containing paracarcinomatous tissues. Paracarcinomatous tissue is taken from non-cancerous tissue 1 cm away from the tumor margin. Normal liver tissue excised near the HCC in 14 patients was also reviewed. The age of patients ranged from 18 to 73 years, with a mean age of 44.4 years (± 11.0 years [SD]). Of the 113 patients, 78 were men and 35 were women.

Follow-up information about the postoperative clinical course of patients was available from outpatient medical records, telephone calls, or letters. Follow-up was successfully completed in 91.2% of the patients for a median of 2 years (range, 3 months-7 years). Intraoperative therapy was not used in any patient. Overall survival time was calculated as the period from surgery until death.

All tumor samples had been fixed in 10% formalin and then embedded in paraffin. Serial 4-μm sections obtained from each specimen were subjected not only to routine hematoxylin and eosin staining to determine their clinicopathological features such as venous invasion, capsule formation, Edmondson's grade, cirrhotic nodule but also to immunohistochemical staining to detect antigens to CD105, CD34, and vascular endothelial growth factor (VEGF), a known marker of angiogenesis.

### Microvessel counting and VEGF scoring

Immunohistochemical staining for CD34, CD105, and VEGF was performed using a sensitive streptavidin-biotinylated horseradish-peroxidase complex method. The antibodies used in our study were purchased as following: mAb to CD105(clone SN6h, NEOMARKERS), mAb to CD34(clone QBEnd/10, NEOMARKERS), or mAb to VEGF(clone JH121, NEOMARKERS)

All of the procedures were performed according to the manufacturer's protocol. Briefly, deparaffinized and rehydrated tissue sections were incubated in 3% hydrogen peroxide diluted with methanol for 30 minutes to block the endogenous peroxidase activity. Antigen retrieval was performed by first pretreating sections in a microwave oven or incubating them in 0.1% trypsin for 10 minutes at 37.0°C, after which nonspecific immunoreactivity was blocked by incubating the tissue in normal goat serum at room temperature for 5 minutes before application of the mAb to CD105(clone SN6h, NEOMARKERS), mAb to CD34(clone QBEnd/10, NEOMARKERS), or mAb to VEGF(clone JH121, NEOMARKERS), which were diluted with phosphate-buffered saline (PBS; 0.01 M, pH7.2) at a ratio of 1:100 for 1 h at 37°C.

The sections were then washed in PBS and sequentially incubated in biotinylated goat anti-mouse immunoglobulin G and a streptavidin-biotinylated horseradish- peroxidase complex, according to the manufacturer's instructions (ABC kit; Boster Ltd, Wuhan, China). The staining was performed by immersing slides in 0.05% 3,3' -diaminobenzidine tetrahydrochloride. All tissue sections were counterstained with hematoxylin, dehydrated, and mounted. PBS, substituted for the primary antibody, was used as a negative control.

The immunostained sections are scanned at low magnification (×40), and three tumor area with the highest density of distinctly highlighted microvessels ("hot spot") within each section were selected for quantitation of angiogenesis. All brown-stained endothelial cell or endothelial cell cluster, which was clearly separate from connective tissue elements, was considered a microvessel. Counting was performed at a high magnification(× 200). The mean counts for each specimen were recorded as the CD34-MVD or the CD105-MVD [30]. VEGF expression was evaluated by the scoring method reported by Shimizu [31]. The immunohistochemical results for VEGF are classified as follows: -, no staining; +, weak staining; ++, strong staining. Microvessel counting and VEGF scoring were performed simultaneously by two independent investigators without any knowledge of the characteristics of the patients from whom the specimens came.

### Statistical analysis

MVD values were compared between groups categorized by their various clinicopathological features by using the Student's *t *test. The postoperative survival rate was analyzed by the Kaplan-Meier method, and the differences were assessed by the log-rank test. A multivariate analysis of the possible prognostic markers was performed using Cox's regression model. Differences were considered significant when P was less than 0.05. All statistical manipulations were performed using the SPSS for Windows system (version 10.0; SPSS Institute, Cary, NC).

## Results

### CD105 and CD34 expression

**Figure 1:**
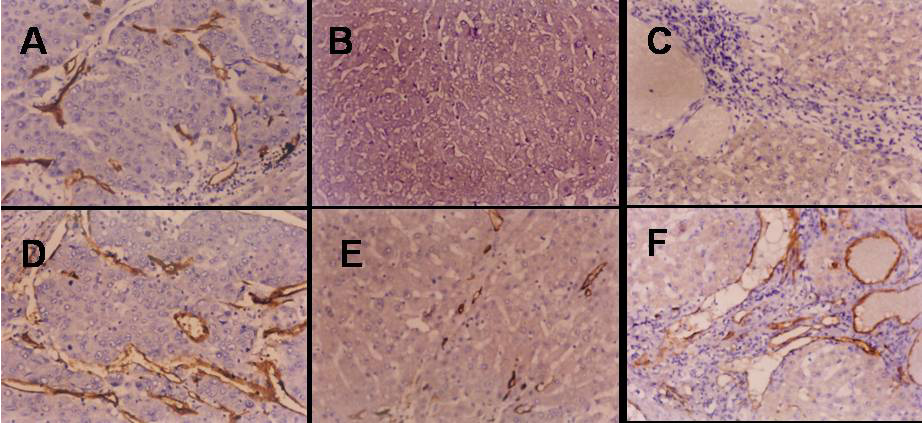
*A*, Positive staining for CD105 in HCC tissue (avidin-biotin-peroxidase complex, original magnification × 200). *B*, Negative staining for CD105 in paracarcinomatous liver tissue (avidin-biotin-peroxidase complex, original magnification × 200). *C*, Negative staining for CD105 in normal liver tissue (avidin-biotin-peroxidase complex, original magnification × 200). *D*, Positive staining for CD34 in HCC tissue (avidin-biotin-peroxidase complex, original magnification × 200). *E*, Positive staining for CD34 in paracarcinomatous liver tissue (avidin-biotin-peroxidase complex, original magnification × 200). *F*, Positive staining for CD34 in normal liver tissue (avidin-biotin-peroxidase complex, original magnification × 200).

**Figure 2:**
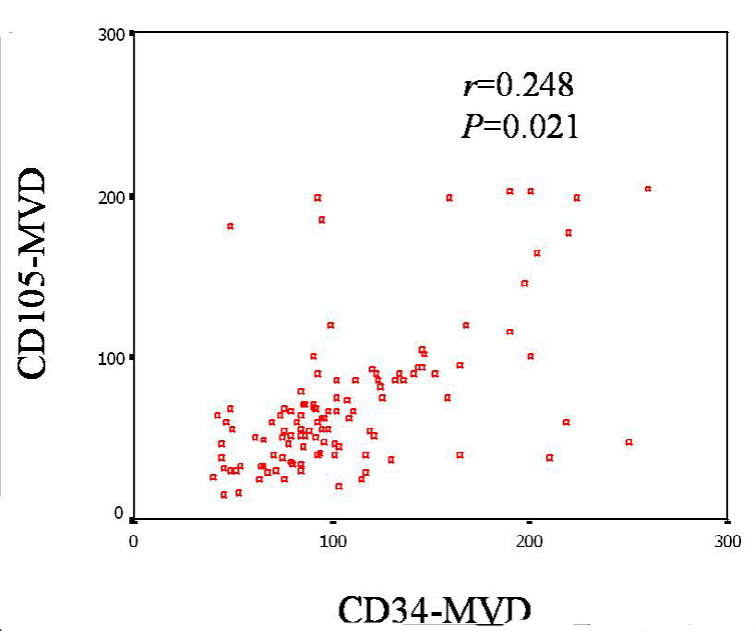
Correlation between CD34-MVD and CD105-MVD.

CD105 was not expressed in the vascular endothelial cells of the 14 normal liver tissues and the paracarcinomatous liver tissue in any of the 113specimens (Fig. 1A–C) but was expressed in the vascular endothelial cells of HCC tissue of all specimens. In contrast, CD34 was expressed in the following proportions of specimens of the vascular endothelial cells of normal liver tissue, paracarcinomatous tissue, and HCC tissue: 86.7%, 93.8% and 100%, respectively (Figure 1, D-F). The CD105-MVD and CD34-MVD were 71.7 ± 8.3 (SD) and 106.3 ± 10.4 (SD), respectively. There was a significant correlation between the CD105-MVD and the CD34-MVD (r = 0.248, P = 0.021; Fig. 2).

### MVD and VEGF expression

**Figure 3:**
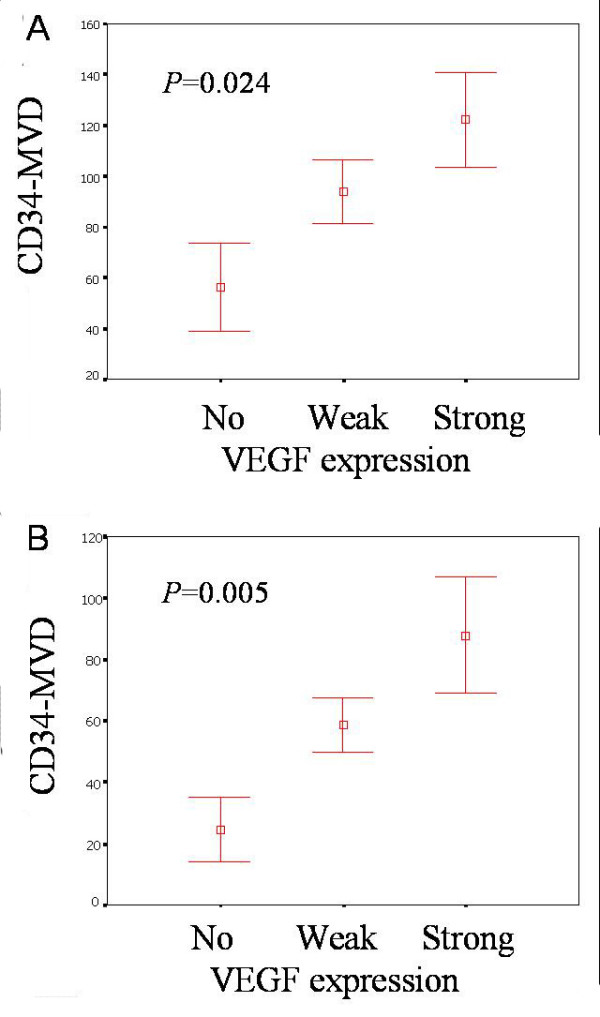
*A*, correlation between VEGF expression and CD34-MVD. *B*, correlation between VEGF expression and CD105-MVD.

The CD105-MVD and CD34-MVD markedly increased with enhanced VEGF expression (*P *= 0.005 and *P *= 0.024, respectively) (Fig.3), indicating that both are markers of the degree of angiogenesis.

### MVD and clinicopathological features

The correlation between CD105-MVD and microscopic venous invasion was significant (*r *= 0.254, *P *= 0.018). We did not, however, find any significant correlation between CD105-MVD and liver cirrhosis, tumor diameter, capsule formation, Edmondson's grade, or preoperative α-fetoprotein concentration (Table 1). There was also no significant correlation between CD34-MVD and any of these clinicopathological features. Thus, CD105 appears to be both specific and sensitive as a marker of angiogenesis.

### MVD and postoperative prognosis

**Figure 4:**
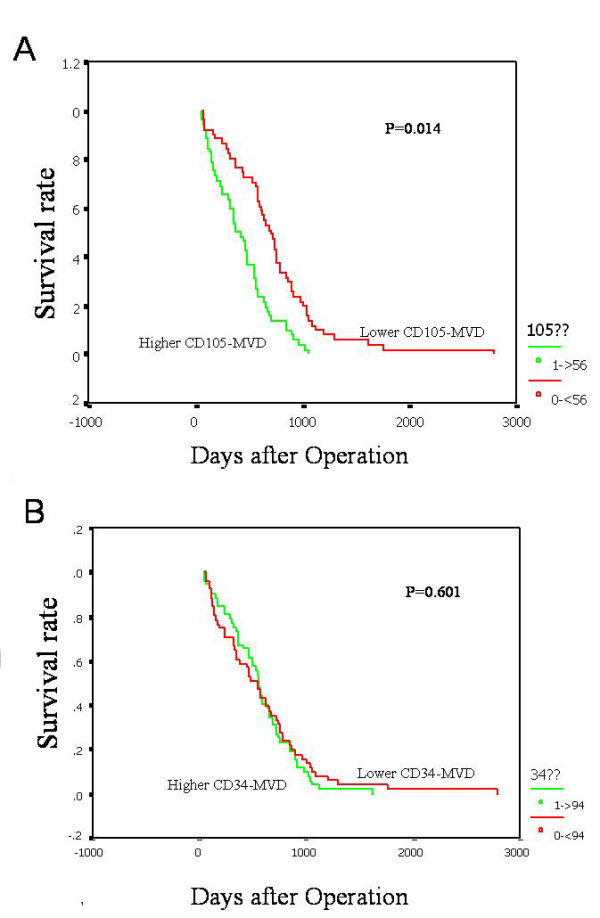
Postoperative overall survival rate of patients with HCC. The comparison shown is according to the MVD determined with an anti-CD105 mAb *A *or an anti-CD34 mAb *B*.

The 1-year, 2-year, and 3-year overall survival rates of all the HCC patients in our study were 65.1%, 30.1%, and 5.8%, respectively. The 2-year survival rate of HCC patients with a lower CD105-MVD (< 56, the median value) was 47.1%, which was significantly higher than the 13.5% rate of HCC patients with a higher CD105-MVD (*P *= 0.014; Fig. 4A). Although the 2-year survival rate (33.3%) of HCC patients with a lower CD34-MVD (< 94)) was also higher than that of HCC patients with a higher CD34-MVD (26.9%), the difference was not significant (*P *= 0.601; Fig. 4B).

### Multivariate analysis of CD105-MVD as a predictive factor

Multivariate analysis showed that a higher CD105-MVD was a significant (P = 0.024) and independent factor predicting a poor prognosis (Table 2). When the CD34-MVD was used in the regression model, however, it failed to show a significant prognostic value (*P *= 0.072).

## Discussion

The high incidence of postoperative recurrence and metastasis has been the major obstacle to further improving the overall survival rate of patients with HCC. Recently, much attention has been paid to the association between angiogenesis and postoperative recurrence or metastasis. Angiogenesis, the formation of new blood vessels from preexisting ones, is essential for any malignant tumor, including HCC, to grow and metastasize; in fact, solid tumors cannot grow beyond 1 to 2 mm in diameter in the absence of angiogenesis. Therefore, the ability to quantitatively distinguish between tumor neovascularization and pre-existing vessels is important in the assessment of tumor angiogenesis. The degree of neovascularization, or MVD, is now generally identified by immunohistochemical staining of endothelial cells with the so-called pan-endothelial cell markers such as CD34, CD31, and von Willebrand factor; however, anti-pan-endothelial antibodies can react with not only newly formed vessels but also normal vessels trapped within tumor tissues. Thus, the MVD identified by anti-pan-endothelial antibodies is not an ideal prognostic marker. It is necessary to find a marker that specifically reacts with the endothelial cells of angiogenic tissue and not with the endothelial cells of most normal tissue.

Endoglin (CD105) is a proliferation-associated and hypoxia-inducible glycoprotein abundantly expressed in angiogenic endothelial cells [32,33]. It is a receptor for the transforming growth factor β superfamily [34,35] and is essential in angiogenesis [36]. Immunohistochemical studies have shown that CD105 is strongly expressed in blood vessels of tumor tissue. The intensity of staining for CD105 is greater in blood vessel endothelia within neoplastic tissue than within normal tissues, indicating that CD105 is a powerful marker of neovascularization in solid malignancies. The MVD determined with the anti-CD105 antibody has been established as an independent prognostic indicator, with increased values correlating with shorter survival times.

To test whether the anti-CD105 mAb reacts only with new vessels in HCC tissues, we used immunohistochemical staining to determine the CD105 expression in tumor specimens from 113 patients with HCC and correlated our findings with available follow-up information about the patients. We found that the anti-CD105 mAb was an ideal marker to distinguish between the microvessels of tumor and normal tissues and that CD105-MVD was significantly correlated with prognosis in patients with HCC. In addition, our finding of the expression of CD34 in both normal and tumor tissue indicated that it is not a reliable marker for HCC.

When we studied the correlation between the expression of MVD and VEGF, a known marker for angiogenesis, we found that although CD34-MVD was significantly correlated with VEGF expression (*r *= 0.243), CD105-MVD was more closely correlated (r = 0.300). This finding further showed that the anti-CD105 antibody is superior to the anti-CD34 antibody in detecting angiogenesis in HCC. However, because angiogenesis is influenced not only by VEGF, to verify the view of CD105 as a marker, comparative studies need to be performed to evaluate the correlation of the CD34-MVD and the CD105-MVD with other factors that influence angiogenesis.

When we analyzed MVD in conjunction with the clinicopathological characteristics of the HCC tumors, we found that the CD105-MVD, but not the CD34-MVD, was strongly correlated with the presence of microscopic venous invasion; microscopic venous invasion has been shown by other studies to be one of the most accurate prognostic indicators of postoperative recurrence and metastasis in HCC [38-40]. This correlation also suggested that anti-CD105 antibodies react with new vessels in HCC tissue more specifically than do anti-CD34 antibodies. We found no significant correlation between CD105-MVD and liver cirrhosis, tumor diameter, capsule formation, Edmondson's grade, or preoperative α-fetoprotein concentration, although other investigators have [41-44]. This discrepancy suggests that our results, although sensitive, are potentially not specific, and further studies will need to be done to confirm the specificity we observed for the marker.

Kaplan-Meier analysis showed that the CD105-MVD, but not the CD34-MVD, was significantly correlated with the 2-year survival rate, and multivariate analysis showed that CD105-MVD, but not CD34-MVCD, was a significant and independent prognostic factor, which was consistent with the results of retrospective studies of other tumors.

Another major benefit of our study is that it identified a CD-105 as a potential target of therapy in HCC. Because angiogenesis is essential for metastasis and recurrence, destroying the tumor-associated microvasculature without severely damaging normal tissues or causing major adverse effects is an appropriate goal for biological therapy. An appropriate proliferation-associated antigen located on the endothelial cells of tumors could be an ideal target for this antiangiogenic therapy and have negligible adverse effects [45-50]. As demonstrated in our study, the anti-CD105mAb specifically reacted with the neovascular endothelial cells of HCC only. Thus, CD105 may be useful not only for its prognostic ability but also in the treatment of patients with HCC.

## Conclusion

In summary, the anti-CD105 mAb proved to be an ideal means to quantify new vessels in HCC, and CD105-MVD was a significant and independent prognostic factor for HCC. We would like to add the cautionary note that a proportion of our patients who were alive despite a relapse had a low MVD and, after a longer follow-up period, might be expected to do poorly. Therefore, our findings need to be confirmed in larger cohorts of patients with a longer follow-up period.

## Competing interests

The author(s) declare that they have no competing interests.

## Authors' contributions

Yang LY and Lu WQ conceived of the study and carried out design and immunohistochemical staining. Huang GW reviewed the all pathological date. Wang W collected the following up information and carried the data analysis and helps to draft the manuscript. All authors read and approved the final manuscript

## Pre-publication history

The pre-publication history for this paper can be accessed here:



## Figures and Tables

**Table 1:** Correlation between microvascular density and clinicopathological features of 113 tumors

Feature	No.	Microvascular density			
		
		CD105*	*P *value	CD34*	*P *value
Venous invasion					
No	52	57.04 ± 5.88	0.032	95.71 ± 5.50	0.392
Yes	61	84.19 ± 3.73		115.33 ± 4.39	
Capsule formation					
Negative	32	56.30 ± 2.39	0.114	90.51 ± 7.69	0.130
Positive	81	77.78 ± 1.88		112.54 ± 4.97	
Edmondson's grade					
I--II	41	63.96 ± 4.55	0.281	105.38 ± 6.17	0.970
III--IV	72	76.11 ± 8.63		106.82 ± 8.22	
Cirrhotic nodule					
≤3 mm	33	69.71 ± 7.60	0.822	99.04 ± 5.78	0.461
> 3 mm	80	72.52 ± 9.78		109.29 ± 8.77	
Serum α-fetoprotein level					
≤20 ng/ml	21	63.60 ± 7.90	0.384	102.92 ± 2.46	0.738
> 20 ng/ml	92	73.55 ± 9.11		107.07 ± 2.78	

*Values are means ± SD.

**Table 2:** Multivariate analysis of prognostic factors in hepatocellular carcinoma

Factor	β	*P *value	Exp(B)
Analysis A			
Venous invasion	0.563	0.041	1.852
Capsule formation	0.035	0.241	0.923
Edmondson's grade	0.272	0.364	1.427
Cirrhotic nodule	0.195	0.148	1.327
Serum AFP	0.047	0.286	0.814
CD34-MVD	0.381	0.072	1.314
Analysis B			
Venous invasion	0.371	0.047	1.536
Capsule formation	0.013	0.191	1.043
Edmondson's grade	0.254	0.305	1.094
Cirrhotic nodule	0.093	0.174	0.978
Serum AFP	0.011	0.197	1.146
CD105-MVD	0.740	0.024	2.270

Abbreviations: AFP, α-fetoprotein level; MVD. Microvascular density.
